# CEACAM1: Expression and Role in Melanocyte Transformation

**DOI:** 10.1155/2016/9406319

**Published:** 2016-08-24

**Authors:** Gabriela Turcu, Roxana Ioana Nedelcu, Daniela Adriana Ion, Alice Brînzea, Mirela Daniela Cioplea, Lucia Beatrice Jilaveanu, Sabina Andrada Zurac

**Affiliations:** ^1^Colentina Clinical Hospital, 1st Dermatology Department, “Carol Davila” University of Medicine and Pharmacy, 020125 Bucharest, Romania; ^2^Department of Pathophysiology II, Clinical Department No. 2, “Carol Davila” University of Medicine and Pharmacy, 021105 Bucharest, Romania; ^3^Pathology Department, Colentina Clinical Hospital, “Carol Davila” University of Medicine and Pharmacy, 020125 Bucharest, Romania; ^4^Department of Medicine, Section of Medical Oncology, Yale University School of Medicine, New Haven, CT 208028, USA

## Abstract

Metastases represent the main cause of death in melanoma patients. Despite the current optimized targeted therapy or immune checkpoint inhibitors the treatment of metastatic melanoma is unsatisfactory. Because of the poor prognosis of advanced melanoma there is an urgent need to identify new biomarkers to differentiate melanoma cells from normal melanocytes, to stratify patients according to their risk, and to identify subgroups of patients that require close follow-up or more aggressive therapy. Furthermore, melanoma progression has been associated with the dysregulation of cell adhesion molecules. We have reviewed the literature and have discussed the important role of the expression of the carcinoembryonic antigen cell adhesion molecule 1 (CEACAM1) in the development of melanoma. Thus, novel insights into CEACAM1 may lead to promising strategies in melanoma treatment, in monitoring melanoma patients, in assessing the response to immunotherapy, and in completing the standard immunohistochemical panel used in melanoma examination.

## 1. Introduction

Metastases represent the main cause of death in melanoma patients. Metastatic melanoma treatment is unsatisfactory, even with the current optimized targeted therapy (BRAF-oncogene inhibitors) or immune checkpoint inhibitors (anti-cytotoxic T-lymphocyte-associated antigen 4 (anti-CTLA4), anti-programmed death-1 protein (anti-PD1), and programmed death ligand-1 protein (anti-PDL1)). To date, lactate dehydrogenase (LDH) is the only serum biomarker used in clinical practice for melanoma; however, the sensitivity and specificity of LDH in predicting metastatic recurrence are low. Considering both the relative unsatisfactory potential of LDH as a biomarker and the poor prognosis of advanced melanoma, numerous attempts by our group and others have been made to identify new biomarkers to stratify patients according to their risk, thus identifying subgroups of patients that require closer follow-up or more aggressive therapy [1]. New blood biomarkers panels including CEACAM are tested, in order to complete laboratory and imaging recommendations for monitoring patients with melanoma [2]. Markel et al. report higher levels of serum CEACAM1 for melanoma patients compared with healthy donors [3].

Melanoma is an immunogenic tumor in which malignant melanocytes present high rate of DNA mutation and express tumoral antigens that can elicit multiple immune responses [4–9]. Dysregulation of cell adhesion molecules has been associated with disease progression in melanoma [10]. Thus, novel insights into carcinoembryonic antigen cell adhesion molecule 1 (CEACAM1) may lead to promising strategies in melanoma treatment.

## 2. CEACAM1 Structure

CEACAM1 (also referred to as C-CAM, biliary glycoprotein BGP, and CD66a) is a complex glycoprotein linked to the cell membrane by a carboxy-terminal transmembrane anchor; it belongs to the carcinoembryonic antigen (CEA) family of the immunoglobulin superfamily. So far, six other CEACAM glycoproteins have been described (CEACAM3–CEACAM8), all of them being characterized by several domains: an N-domain, a membrane IgV-related domain, and one/multiple IgC2-related domains. CEACAM1 N-domain may bind homophilically with CEACAM1 and heterophillically with CEA or other CEACAMs, thus acting as cell-cell adhesion molecule [11, 12].

CEACAM1 is encoded on chromosome 19q13.2 and is expressed on several types of cells such as epithelial cells (apical pole of epithelial cells of the gastrointestinal tract, esophageal glands, duodenal Brunner glands, intestinal and colonic superficial epithelial cells, epithelial cells of the gall bladder, bile ducts, pancreatic ducts, mammary ducts, epithelial cells of the endometrium, renal tubular epithelium, prostatic glands, extravillous (intermediate) trophoblast, etc.), endothelial cells, B and T lymphocytes, natural killer cells, and myeloid cells [13–19].

## 3. CEACAM1 General Biologic Functions

The expression of CEACAM1 mediates intercellular protein interactions and intracellular signaling during inflammation, microbial and viral infection, angiogenesis, cancer progression, and metastasis [20].

The role of CEACAM1 in inflammation is complex due to its alternate splicing and generation of various isoforms; in humans, eight transmembrane isoforms are present (including CEACAM1-3L, CEACAM1-4L, CEACAM1-3S, and CEACAM1-4S). In T lymphocytes CEACAM1 splice variants with long cytoplasmic tails associate with inhibitory function both* ex vivo* and* in vivo* by inhibiting the T-cell receptor CD3 complex. Moreover, these splice variants also enhance fibrinogen adhesion via Fc receptor and *β*2 integrin [12, 15, 21–24]. CEACAM1 is involved in modulation of innate and adaptive immune responses, via its expression on B and T lymphocytes, natural killer cells, and myeloid cells. CEACAM1 has a role in T cell inhibition through direct T cell receptor cross-linking and binding of* Neisseria* opacity associated proteins; CEACAM1 inhibition of NK cells is independent of the major histocompatibility complex class I and dependent on the cytosolic tail [25].

CEACAM1 regulates angiogenesis by targeting several processes such as chemotaxis, endothelial cells proliferation, and tube formation; its biologic functions are additive to vascular endothelial growth factor (VEGF) effects, as demonstrated by CEACAM1 upregulation induced by VEGF as well as* in vitro* obstruction of VEGF-induced tube formation by CEACAM1. Several tissues overexpress CEACAM1 in endothelial cells such as normal or pathological nontumoral highly proliferating tissues (normal and/or pregnant endometrium, granulation tissue) or tumor tissues (such as solid human tumors) [19, 26].

CEACAM1 also intervenes in insulin action regulation increasing the clearance of insulin through the stimulation of receptor-mediated insulin endocytosis and degradation [27].

CEACAM1 is used by various pathogens to adhere to eukaryotic cells. For instance,* Haemophilus influenzae*,* Moraxella catarrhalis*,* Escherichia coli*, and various species of* Neisseria and Salmonella* use the N-domain of CEACAM1 as a microbial receptor in human granulocytes and epithelial cells. Several viruses such as coronaviruses mouse hepatitis virus type 2 and severe acute respiratory syndrome coronavirus (SARS-CoV) use a soluble form of CEACAM1a to enter host cells [12, 28, 29].

Furthermore, CEACAM1 is also involved in apoptotic control by two mechanisms, the cleavage of CEACAM1-4L isoform intra- and extracellularly and caspase activation [30–32].

## 4. CEACAM1 Expression in Cancer

High expression levels of CEACAM1 have been detected in melanoma [33], adenocarcinomas [34], and small cell lung cancers [35] while lower levels of CEACAM1 have been detected in colon [36], prostate [37], and breast cancers [38].

In melanoma, through homophilic interactions, CEACAM1 inhibits natural killer (NK) cell activity and effector functions (such as cytotoxicity and interferon gamma (IFN*γ*) release) of tumor-infiltrating lymphocytes (TILs). Upregulation of CEACAM1, induced by IFN*γ* on melanoma cells that survive TIL-mediated attack, renders these cells even more resistant [16, 25, 39–41]. CEACAM expression in melanoma has often been noted in the invading part of the tumor [42] and has been associated with increased melanoma cell invasion and migration [43]. CEACAM also interferes with cell-matrix adhesion and enhances cell motility by modulating N-cadherin, an intercellular adhesion glycoprotein and epithelial-mesenchymal transition marker [44].

Recent studies evidenced the correlation between CEACAM1 expressions in melanoma cell lines and melanoma tissue with microphthalmia-associated transcription factor (MITF) [45]. MITF modulates proliferation and invasion in melanoma.

Mechanisms involved in CEACAM1 and MITF correlation in melanoma may involve sex determining region Y [SRY] related HMG-box 9 (SOX9); SOX9 is a transcription factor involved in chondrogenesis and sex determination in embryo [46]; its function in normal melanocytes is upregulation of melanin synthesis in melanocytic cells after ultraviolet B (shortwave) rays (UVB) exposure by increasing MITF, dopachrome tautomerase (tyrosine-related protein 2), and tyrosinase expression [47], while increased expression of SOX9 in melanoma inhibits tumor cell proliferation by binding p21 directly or via MITF [48, 49]. Whether SOX9 directly or indirectly regulates CEACAM1 expression is still a matter of debate with different authors obtaining opposing results, possibly due to differences between the types of cells studied (colon epithelium versus melanoma cells lines) and the differences of the roles fulfilled by CEACAM1 in colon carcinoma (tumor growth suppressor) and melanoma (tumor aggressiveness promoter) [50–52]. Also, MITF binds the CEACAM1 promoter on an M-box motif, thus directly stimulating CEACAM1 expression [45].

Moreover, CEACAM1 induces Sox-2 overexpression, which in turn induces *β*-catenin expression, a mechanism involved in acquiring tumor aggressiveness. Sox-2 increases invasiveness of melanoma cells; it is upregulated by CEACAM-1L and has a similar progressive upregulation from 14% in nevi to 80% in metastatic melanoma [53–55].

## 5. CEACAM1 in Benign Melanocytic Lesions

CEACAM1 is not present on the surface of normal melanocytes [43]. There is a lack of data regarding CEACAM1 expression in melanocytic nevi. Gambichler et al. performed an immunohistochemical study, analyzing the expression of CEACAM1 in benign and malignant cutaneous tumors and in normal peritumoral skin: benign nevi (42 cases), dysplastic nevi (22 cases), thin superficial spreading melanomas (21 cases), and thick superficial spreading melanomas (21 cases). The results were reported as percentages of positively stained melanocytes in studied lesions. Median CEACAM1 expression was 1% in benign nevi, 9.6% in dysplastic nevi, 18% in thin superficial spreading melanomas (Breslow tumor thickness < 1 mm), and 74% in thick superficial spreading melanomas (Breslow tumor thickness > 1 mm) (*p* < 0.0001). Compared with benign nevi, median expression of CEACAM1 was significantly increased in both thin and thick superficial spreading melanomas and was not significantly increased in dysplastic nevi. Compared with dysplastic nevi, median expression of CEACAM1 was significantly increased in thick superficial spreading melanomas. In peritumoral skin, melanocytes were not immunohistochemically reactive [56].

CEA family expression was immunohistochemically assessed in different subtypes of melanocytic nevi: 5 junctional acquired nevi, 9 compound acquired nevi, 31 intradermal acquired nevi, 14 compound congenital nevi, 4 intradermal congenital nevi, and 20 blue nevi. The authors used a panel of monoclonal and polyclonal antibodies that recognize different epitopes of CEA and CEA-related molecules, including CEACAM1; none of the antibodies used in the study recognized only CEACAM1. The results of the study revealed an increased expression of CEA glycoprotein family in the various types of analyzed nevi, excepting blue nevi. The negative staining was also observed in normal melanocytes [57]. The carcinoembryonic antigen family is expressed in melanocytic nevi, in a similar pattern in acquired and congenital ones, and it is not present in blue nevi that represent neural crest melanocytes that failed to reach the epidermis during embryological migration [57].

*In vitro* investigation evidenced that invasion and migration of melanocytic cells that express CEACAM1 are enhanced. The authors studied melanocytic MEL6 cell line invasive capacity, after cells were transfected with CEACAM1. CEACAM1 transfected cells showed higher invasive and motility properties compared to untransfected cells [43].

## 6. CEACAM1 in Primary Melanoma

Gamblicher studied 42 cases of superficial spreading melanoma (SSM), using 2 different antibodies that revealed membranous and cytoplasmic staining of tumor cells. CEACAM1 expression was significantly higher in SSM compared to benign nevi, and the authors noticed a progressive increase from benign nevi, dysplastic nevi, and thin SSM to thick SSM. The authors highlighted a significant positive correlation between CEACAM1 expression and Breslow tumor thickness and Clark level of superficial spreading melanomas [56].

In 2002 Thies et al. evaluated 100 cases of primary melanoma and 40% expressed CEACAM1, the staining being more intense in the invasive part of the lesion. Out of the 40 cases of CEACAM1 positive melanomas, 28 had metastasized while out of the 60 cases of CEACAM1 negative melanomas, only 6 had metastasized. Vessel staining was identified in only 7 patients and there was no association between CEACAM1 expression in microvessels and prognosis [42].

In activated human tumor-infiltrating lymphocytes derived from patients with melanoma, all cells express CEACAM1, with inhibitory effects following homophilic interaction [41, 42].

The authors concluded that CEACAM1 expression in melanoma cells was an independent factor (regardless of the ulceration status, mitotic rate, and tumor thickness) for the risk of metastasis with a predictive value superior to that of tumor thickness. For patients with identical parameters in American Joint Committee on Cancer (AJCC) classification, CEACAM1 status provides a more accurate predictive estimation [42].

Studying the essential interplay between melanoma and tumor-infiltrating lymphocytes, Markel et al. coincubated melanoma cells with tumor-infiltrating cells* in vitro*. Results of the experiment showed a progressive increase in CEACAM1 expression on melanoma cells that resisted the attack of infiltrating lymphocytes. Increased CEACAM1 expression was dependent on the presence of interferon gamma [25].

## 7. CEACAM1 in Metastatic Melanoma

Ortenberg et al. showed that 89% of metastatic cutaneous melanoma lesions express CEACAM1, and its expression increases during tumor progression [53].

Khatib et al. first evaluated CEACAM1 expression in 79 cases of primary uveal melanomas and 21 metastases in the most frequently affected organ, the liver. CEACAM1 was identified in 45% of the primary uveal melanomas and in 81% of liver metastases, similar to CEACAM1 expression in cutaneous melanoma and its corresponding metastases. The presence of CEACAM1 on the primary tumor did not correlate with the development of metastases or survival [58].

*In vivo* studies showed that CEACAM1-L overexpression promotes xenograft tumorigenicity of melanoma [53]. CEACAM1 directly inhibits activated NK and T lymphocytes and this results in an increase of its expression in melanoma cells that survive an* in vitro* immune attack, which can further inhibit new immune cells [41, 42, 59, 60].

*In vitro* studies evidenced another possible mechanism responsible for these data: the expression of CEACAM1-L in melanocytic cells (CEACAM1 negative MEL6 melanocytic cell line) and melanoma cell (CEACAM1 negative MV3 melanoma cell line) increased the invasive and the migratory properties of melanocytic and melanoma cells, by interaction with integrins [43]. Cell migration was also modulated by interaction of CEACAM1-L and the protein filamin A, which reduce the migratory potential. The authors evidenced that CEACAM1-L, in addition to stimulation of migration, could also inhibit cell migration [61].

In a small series of 13 patients, Zippel et al. noticed a borderline significant increase in the membrane staining from primary lesions to lymph nodes and distant metastases [62].

In current practice immunohistochemical staining for S100, melanA, and human melanoma black 45-HMB45 is used to identify melanoma cells in clinically negative sentinel lymph nodes, with 10–20% improved rate of detection compared to hematoxylin and eosin (HE) staining alone. Immunohistochemical marker sensitivity and specificity for detection of melanoma cells in primary melanomas, sentinel lymph nodes, and distant metastases were studied by Thies et al. [33]. Expression of cell adhesion molecules CEACAM1 and L1 was analyzed and compared to expression of standard markers MelanA, S100, and HMB45 in 67 cases of primary melanomas, 40 cases of sentinel lymph nodes, 35 cases of distant metastases, and 12 cases of benign nevi. The authors compared the sensitivity of CEACAM1 and L1 markers to MelanA, S100, and HMB45 and describe results similar to other studies reporting an 87–93% sensitivity in primary melanomas and 60–95% in their corresponding metastatic lymph nodes [63]. The authors also compared two different antibodies for CEACAM1 and highlighted the superior sensitivity of monoclonal antibody 4D1/C2 to the commercial NCL-CD66a. Regarding the specificity of immunohistochemical markers for melanoma cells, a relevant observation was described; CEACAM1 and L1 are highly specific for melanoma cells, while MelanA, S100, and HMB45 are not. In this context, benign nevus cell inclusions in the capsule of sentinel lymph nodes could lead to false positive diagnosis of melanoma metastases [33].

Antibodies 4D1/C2 against CEACAM1 have a higher specificity and sensitivity for melanoma cells in lymphatic and hematogenous metastases and can be added to the standard panel of antibodies [33, 64].

In a study from 2012 Ortenberg et al. found positive staining for CEACAM1 in 89% of the metastatic melanoma and in CD8 lymphocytes surrounding the metastases [65]. The wide distribution of CEACAM1 in metastatic melanoma qualifies it for targeted therapy, alone or in combination.

The new immune checkpoint inhibitors, anti CTLA4 and anti PD1, are not tumor-specific and are sometimes associated with severe immunologic side effects.

*In vitro* and* in vivo* studies on melanoma xenografts show that antibodies built to target the extracellular portion of CEACAM1 and to block the N-domain of CEACAM1 (MRG1, a murine IgG1 monoclonal antibody against human CEACAM1) did not influence the proliferation rate but facilitate melanoma cell elimination by T cells and have no agonistic effect. Regarding the safety concerns about MRG1 antibody's effect on normal epithelial cells that express CEACAM1 or the effect of activated T cells on normal CEACAM1 positive cells and autoimmune events, it did not directly affect CEACAM1 positive cells and it did not induce nonspecific activation of T cells [53]. It is thought that concomitant blocking of CEACAM1 and immunotherapy with adoptive cellular transfer (ACT) might improve the clinical response [41].

Novel specific monoclonal antibodies for melanoma immunotherapy, based on functional blocking of CEACAM1, are now available from cCAM Biotherapeutics (CM24) and Agenus Inc. [65, 66].

A phase I trial (NCT02346955) is going on in 2 centers from the USA and 1 center from Israel and a primary outcome measure will be available after January 2018 [67]. Blocking of CEACAM1 may reverse the inhibitory action on the NK and activated T cells and enhance the antitumoral effect of the endogenous immune system.

## 8. CEACAM1 Serologic Results

CEACAM1 is normally identified in serum from healthy individuals, but at a low level. In melanoma patients, the level of CEACAM1 correlates with the amount of tumor cells that secret CEACAM1 [3].

Patients with melanoma also show a high percentage of CEACAM1 positive lymphocytes in the peripheral blood, which is not secreted and does not influence the total serum level of CEACAM1. In metastatic melanoma, the level of soluble CEACAM1 significantly correlated with LDH level [3].

Currently, LDH is the only serologic biomarker included in AJCC staging system as an important independent prognostic factor in advanced, stage IV melanoma, with a 92% specificity and 79% sensitivity [68, 69]. Egberts and collaborators found S100 calcium-binding protein B (S100B) evaluation superior to LDH in the identification of early distant metastasis [70]. The levels of both markers correlates with poor outcome, shorter disease-free and overall survival [71, 72].

Sivan et al. monitored CEACAM1, S100, and LDH serum level in 49 patients with regional or metastatic disease, prior to and after autologous vaccination [73]. Patients with evidence of disease showed significantly higher levels of CEACAM compared to patients with no evidence of disease or healthy volunteers, reflecting the disease burden. Kluger's group also showed that plasma CEACAM1 is elevated in patients with metastatic melanoma compared to healthy controls and early stage disease [2]. Serum levels of CEACAM1 correlated with S100, disease activity, and overall survival rates. The level of soluble CEACAM1 inversely correlated with time to death from melanoma in patients with active disease. As majority of the patients had normal LDH levels, CEACAM1 value facilitates a more precise prognostic prediction [60, 73].

## 9. CEACAM1 and the Risk of Tumor Recurrence

Another important issue for patients with stage I and II resected melanoma is the follow-up schedule, for which there is no general consensus. Melanoma cells can remain dormant for variable periods of time and valid biomarkers to detect recurrences are not yet available, so early surgical or systemic treatment is delayed. Kluger and colleagues proposed a multiplex, plasma-based protein biomarker panel that included CEACAM, ICAM-1 (intercellular adhesion molecule 1), osteopontin, MIA (melanoma inhibitory activity), GDF-15 (growth differentiation factor 15), TIMP-1 (tissue inhibitor of metalloproteinase 1), and S100B. With a sensitivity of 74%, once validated, this panel test can be used to monitor patients for melanoma recurrences and reduce the amount of unnecessary imaging in asymptomatic patients that have normal marker levels [2].

## 10. Conclusions

The reviewed articles highlighted the important role of CEACAM1 expression in the development of melanoma. CEACAM1 promotes melanoma progression: CEACAM1 enhances migration and invasion of melanoma cells; CEACAM1 impairs the antitumor immune responses of NK and T cells. Mechanisms by which CEACAM1 interacts with melanoma biology are incompletely elucidated.* In vitro* studies revealed the relation between CEACAM1 expression and melanoma cells behavior; increased expression of CEACAM1 in melanoma cell lines amplified the invading capacity of these cells; downregulation of CEACAM1 expression in melanoma cells decreased the expansion ability of cells. On the other hand, CEACAM1 expression modulates melanoma cell escape from immunologic attacks; when melanoma cells and tumor-infiltrating lymphocytes are coincubated* in vitro*, surviving melanoma cells increase CEACAM1 expression, which is dependent on constant presence of interferon gamma. CEACAM1 positive lymphocytes are more abundant in patients with melanoma; melanoma cells possibly transfer CEACAM1 to the attacking lymphocytes, affecting efficient immune reactions. In patients receiving immunotherapy for melanoma using adoptive cell transfer with tumor-infiltrating lymphocytes, downregulation of major histocompatibility complex (MHC) class I is often described; reduced surface expression of MHC class I molecules is seen in melanoma cells overexpressing CEACAM1. CEACAM1 expression correlates with poor prognosis of melanoma patients. Since melanoma is an immunogenic disease, CEACAM1 constitutes an attractive target for immunotherapy.

Correlation between serum CEACAM1 and melanoma progression and survival supports CEACAM1 as a standard biomarker in monitoring melanoma patients and assessing the response to immunotherapy.

Due to the high specificity and sensitivity of antibodies against CEACAM1 in melanoma metastases, it could enhance the standard immunohistochemical panel used in melanoma examination.

Reviewing the literature we have brought into focus a key molecule, CEACAM1, as one of the latest hallmarks of cancer involved in the main mechanisms responsible for increasing the invasiveness of melanocytes. Reports about CEACAM1 immunoexpression are scarce in the literature, but due to its promising potential for assessment, diagnosis, and targeted treatment, we consider that further investigation is warranted to advance current knowledge.

## Abbreviation

anti-CTLA4: Anti-cytotoxic T-lymphocyte-associated antigen 4
anti-PD1: Anti-programmed death-1 protein
anti-PDL1: Programmed death ligand-1 protein
LDH: Lactate dehydrogenase
CEA: Carcinoembryonic antigen
VEGF: Vascular endothelial growth factor
IFN*γ*: Interferon gamma
UVB: Ultraviolet B (shortwave) rays
SOX-2: Sex determining region Y-box 2
AJCC: American Joint Committee on Cancer
HMB45: Human melanoma black 45
S100B: S100 calcium-binding protein B
MHC: Major histocompatibility complex.
